# Languaging psychopathology: neurobiology and metaphor

**DOI:** 10.3389/fpsyt.2024.1320771

**Published:** 2024-02-05

**Authors:** Adnan Syed, Michael S. Jacob

**Affiliations:** ^1^ Mental Health Service, San Francisco Veterans Affairs (VA) Medical Center, San Francisco, CA, United States; ^2^ Department of Psychiatry and Behavioral Sciences, University of California, San Francisco, San Francisco, CA, United States

**Keywords:** neurobiology, chemical imbalance, metaphor, abduction, clinical communication, models of illness, uncertainty tolerance

## Abstract

Explanatory models of the mind inform our working assumptions about mental illness with direct implications for clinical practice. Neurobiological models assert that the mind can be understood in terms of genetics, chemistry, and neuronal circuits. Growing evidence suggests that clinical deployment of neurobiological models of illness may have unintended adverse effects on patient attitudes, public perception, provider empathy, and the effectiveness of psychiatric treatment. New approaches are needed to find a better language for describing (let alone explaining) the experience of mental illness. To address this gap, we draw upon interdisciplinary sources and semiotic theory to characterize the role of metaphor in the conceptualization and communication of psychopathology. We examine the metaphors recruited by contemporary neurobiological models and metaphor’s role in facilitating descriptive clarity or evocative creativity, depending on intention and context. These multiple roles reveal the implications of metaphorical reasoning in clinical practice, including cognitive flexibility, personalized communication, and uncertainty tolerance. With this analysis, we propose a clinical approach that embraces the meta-process of ongoing novel metaphor generation and co-elaboration, or *languaging* metaphors of psychopathology. Our goal is to bring attention to the value of employing ever-evolving, shapeable metaphorical depictions of psychiatric illness: metaphors that enable a capacity for change in individuals and society, reduce stigma, and nurture recovery.

## Introduction

“Metaphorical language depends on non-metaphorical language…the way the art of healing depends on the presence of injury and disease (1).”

In clinical and public health settings, diverse models of the mind are deployed to make sense of human experience and the experiences of mental illness. Despite the emphasis on biological psychiatry in residency training programs, most clinicians are also influenced by non-biological, philosophical, psychological, and folk models adopted from daily life. An even broader range of models is adopted by the general public, including various ideas from popular culture, alternative medical models, religious, and spiritual perspectives. These diverse models serve to organize the richness of experience into something that can be comprehended and communicated. As described in the seminal work of Kleinman and associates on the topic (2), learning about and responding to the explanatory models of illness held by patients provides a crucial opportunity to improve provider-patient communication: to acknowledge differences, align goals, and join together in treatment planning.

In psychiatry, even with the recognition of the importance of explanatory models in the clinical setting, most research has emphasized the scientific validity of specific models. However, in recent decades there has been notable advocacy of explanatory pluralism and attempts to integrate overlapping causal models (3, 4). In line with this, researchers have begun developing formal methods to examine the scientific validity of this pluralistic approach, including efforts to join both biological and non-biological factors into causal explanations of mental disorders (5). Still, reductive biological explanations remain prevalent. This fits the biomedical model that mental disorders *are* brain disorders which are therefore correctable with targeted medications, a concept that in recent years has come under increased scrutiny (6). Though novel nosological efforts to better characterize psychopathology are in active development, such as the Hierarchical Taxonomy of Psychopathology (7), they are not widely adopted by clinicians or scientists compared to the dominant neurobiological paradigms. Moreover, all of these approaches emphasize the technical language of scientific classification, whose intent is for the clinician-scientist to more precisely characterize the nature of illness and provide relevant treatment. However, in the study, application and development of these models, there is less emphasis on the communicative intent and the implications of specific technical language on clinical care.

Despite a growing emphasis on interdisciplinary collaboration and shared decision-making between provider and patient in clinical practice, the psychiatrist is still largely seen as the authority on the mind and its models (8–10). This power differential between provider and patient is challenged by competing cultural frames and narratives that may be discordant between biologically-oriented clinicians and their diverse patient populations (11). In fact, the difficulty in establishing shared models of mental illnesses is compounded by differing beliefs among mental health providers themselves (12). Further complicating these efforts, there remains a broadly incomplete account of the varieties of possible psychiatric disorders across cultures, as even the core issue of what is “abnormal” or a “malfunction” itself “shifts its character from culture to culture” (13). The complexity of intersecting personal, public, and scientific contexts in which the mind is considered requires examination of how neurobiological models are conveyed and understood by patients and clinicians.

In the following conceptual analysis, we first review the shortcomings and potential harm associated with neurobiological models of illness. We then interpret these findings through the lens of metaphorical reasoning, considering the ways in which prevalent explanatory descriptions of “chemical imbalances,” “circuits,” or “networks” may create dissonance with the lived experience of mental life and mental illness. Further, we consider that these dominant neurobiological metaphors and their elicited associations risk misunderstanding and stasis, potentially precluding both expressions of individual subjectivity that support healing on the part of the patient as well as novel forms of clinical reasoning on the part of the clinician. Metaphor has been argued to be central to thought itself (14–16), with metaphors mediating and shaping patients’ experience of physiologic pain and psychiatric illness (17, 18). We extend this position to propose that, rather than clinicians simply using more or different specific metaphors, it is the process of *languaging* metaphor – that is, co-elaborating metaphors through creative, associative dynamics – that may evoke new understandings.^1^ Through co-generation of new metaphors, patient and clinician can develop a malleable and shared “living language” to create opportunity for change. To demonstrate the reasoning behind this framework, we begin by first exploring the historic and current state of dominant neurobiological explanations of psychopathology.

## Neurobiological models in clinical practice

Neurobiological explanations of mental illness have been heralded as the future of psychiatry, with neuroscientific advances promising to reveal the mechanistic underpinnings of psychiatric disorders, and thereby unlock novel treatments (21, 22). Furthermore, the promotion of neurobiological models has had the laudable goal of reducing the stigma of mental illness by redirecting blame to a biogenetic pathogenesis (23). However, it is increasingly apparent that the widespread adoption of neurobiological explanations in the clinic presents new challenges and shortcomings (24, 25). Recent evidence suggests that neurobiological models may be ineffective at reducing blame and are moreover associated with heightened perceptions of dangerousness, desire for increased social distance, and pessimism regarding the potential for recovery (26–30). These findings are consistent across multiple meta-analyses, with even stronger associations present when examining experimental (rather than correlational) studies. Worryingly, neurobiological explanations have furthermore been found to reduce clinician empathy across a series of studies (31). As to why this pattern is seen, one proposed hypothesis is that the impact of neurobiological explanations may act through two facets of stigma: attribution of uncontrollability (diminishing individual blame) and psychological essentialism (increasing social stigma) (32). The communication of any model, or indeed no explanation at all, is likely to have both positive and negative effects specific to the target audience, which is influenced by patients’ individual, familial, and broader societal factors (33). However, these findings of worsened stigma highlight how the unexamined communication of existing neurobiological models of the mind may carry unintended yet tangible risks of harm to patients as well as to the general public.

As it stands, few clinicians or patients believe in solely biomedical causes of mental disorders without consideration of other factors. Though contemporary allopathic medical training commonly emphasizes biological pathological mechanisms, psychiatrists often endorse using multiple models of mental illness early in their careers (34, 35). In a series of studies on ontological beliefs about mental disorders, mental health clinicians consistently conceptualized mental disorders as existing along a continuum from having biological origins to psychosocial causes (12). Notably, clinicians who attributed a disorder primarily to a biological cause were less likely to also identify psychosocial factors for that disorder, and vice-versa. This tendency to conceptualize some disorders as diseases of the mind (psychological) and others as diseases of the body (biological) has also been associated with beliefs regarding whether psychotherapy or pharmacotherapy would be most effective for each disorder. Importantly, clinicians’ models in many instances did not match that of the perspectives held by laypeople. For example, laypeople were more likely than clinicians to identify schizophrenia as having psychosocial causes, and laypeople were also much less likely to believe pharmacologic treatments to be helpful for mental disorders. In a separate study that similarly found clinicians employing mind-body dualism, clinicians’ attributions of behavioral problems to psychological versus neurobiological causes were also associated with differing views of patients’ responsibility and blameworthiness for their symptoms (36). Thus, clinicians and patients simultaneously employ a variety of models of the mind with direct implications for clinical reasoning and treatment planning.

Even when examining neurobiological explanations alone, it is important to note that biological models of the mind and mental illness are themselves greatly varied and have evolved rapidly over the last 100 years (30, 37). Furthermore, shifts in biological explanations have often paralleled new therapeutic technologies. Most notably, the advent of 20^th^ century psychopharmacology contributed to the development of the chemical imbalance model. This model was based more on the effectiveness of pharmacology rather than on a mass of underlying bio-pathological evidence, and yet was promoted by psychiatrists and the broader academic community (38). This model then spread rapidly through popular culture and continues to be endorsed by many patients today (39–41), even though it has been challenged by limited supporting evidence (42). Recently, interventional methods such as repetitive transcranial magnetic stimulation (rTMS) may be reinforcing the perception that mental illness results from a brain circuit dysfunction that must be corrected (43). Scientific differences between chemical imbalance and brain circuit models notwithstanding, it remains to be seen how the newer circuit-based descriptions will impact public perception. More broadly, it has been noted that the neurotransmitter-based descriptions and even dimensional conceptualizations of illness, such as the Research Domain Criteria (RDoC), bear resemblance to ancient Greek conceptions of the humors (44). This suggests that, at its core, at least part of the enduring strength of biological models is their appeal to reductionism and the provision of a clear framework for examining health as a process of correcting fundamental imbalances.

## Neurobiology: model or metaphor?

**Figure 1:**
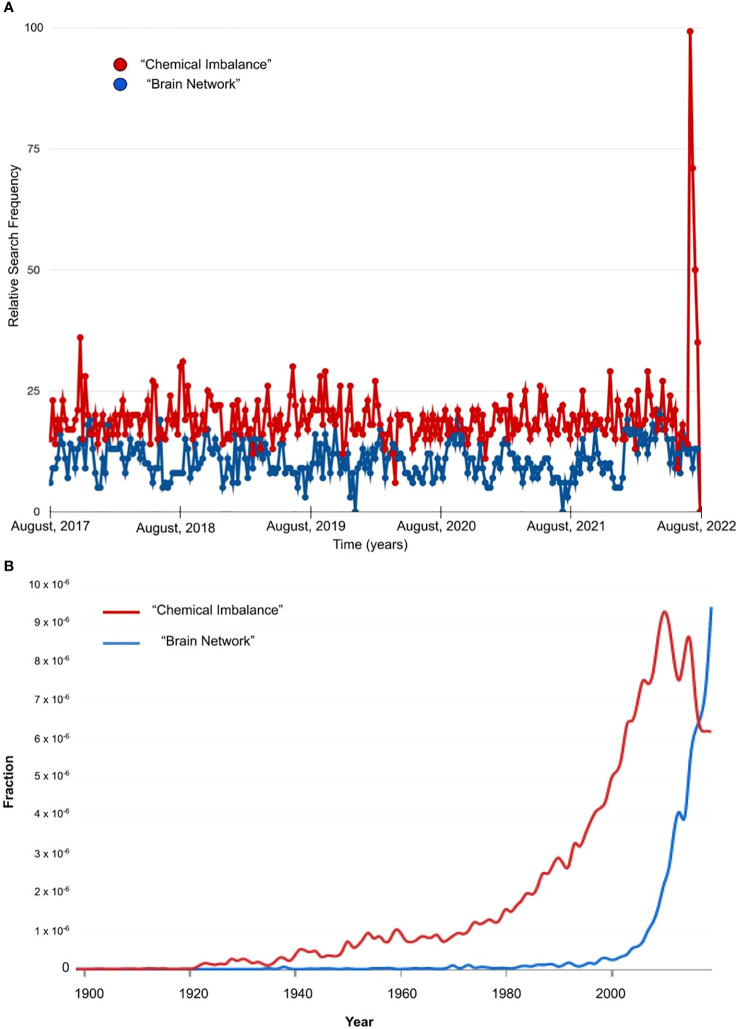
English Language Use of Metaphors for the Brain. (A) Relative search frequency from Google trends for a five year period from 2017-2022 for the terms “chemical imbalance” (red) and “brain network” (blue) in the United States. The spike in search frequency for “chemical imbalance” coincides with media reporting on the Moncrieff et al. (2022) meta-analytic study. (B) Fraction of the English language corpus containing the terms “chemical imbalance” (red) and “brain network” (blue) from Google’s Ngram Viewer during the years 1900-2022.

For all the certainty they may strive to provide, neurobiological models remain in constant evolution, as do their clinical implications and public messaging. Illustrating such a shift in public messaging, media reporting on the Moncrieff et al. (42) study regarding limited supporting data for the serotonin model of depression co-occurred with a marked increase in public online searches related to the term “chemical imbalance,” with a sharp spike particularly in July 2022 (**Figure 1A**). Notably, this search behavior occurred in the context of a relative *reduction* in the number of references to this phrase in published English-language texts – e.g., relative to such phrases as “brain network” (**Figure 1B**). These findings may reflect a larger social trend away from chemical imbalance as an explanatory model, and warrant additional research in this area. Yet just months after wide reporting on a lack of data for the serotonin hypothesis in depression, news outlets also circulated a study with a contrasting message, claiming new evidence for reduced serotonin release in depression (45, 46). As the field shifts toward network-based and computational language and metaphor, it is imperative that clinicians understand how this language impacts patients since it is likely to have an indelible impact on public consciousness, much like the chemical imbalance model.

In addition to uncontrollability and essentialism (32), neuroscientific language may present a unique challenge. As an academic discipline, neuroscience struggles with finding accurate terminology. Neuroscientists regularly use terms such as “encoding” or “representation” to describe the mapping between brain activity and mental experience, despite the fact that such terms are poorly defined (47, 48). Studies may state that they investigate concepts such as “love” and “lying,” but the phenomenon studied may be much narrower than, or miss entirely, the lay meanings associated with such words (49). Speculatively, public understanding of neurobiology may therefore necessitate reliance on simplified models of brain function, or demand comparison to more familiar metaphors such as mechanical descriptions or generic computer software/hardware analogies. This problem is not limited to the non-professional public, since biologists themselves have readily adopted computational models of mental function by utilizing terminology from engineering disciplines (50). The many shortcomings of the computational model of the mind have been raised by neuroscientists, computer scientists, and philosophers alike (51–54). By contrast, there are some who suggest that the brain-computer metaphor is just a matter of “semantics,” since the brain is either literally “a computer” (if one is a computer scientist) or nothing like it (if one is not) (55).

We consider that the semantics of the brain-computer metaphor is actually quite important to the future lives of scientists, clinicians, and the public. Metaphor is central to how we communicate with patients, and arguably also core to how illness itself is conceptualized and experienced by both patients and clinicians. It has even been argued that psychiatry’s theoretical formulations and diagnostic labels began as metaphors (56). Confusing to the public, metaphors in the life sciences may be used to communicate both non-literal and more literal senses, depending on the user, as the brain-computer metaphor highlights. While metaphors may be used in some cases to communicate analogous concepts, retaining elements of theoretical ambiguity and uncertainty, in other instances metaphors (such as the term “cell” itself) may become so repeated and closely associated with a biological phenomenon that they instead serve as a literal, technical, and highly specified term for scientists (57). These highly-specified metaphors may therefore carry entirely different connotations for scientists and laypeople, risking misunderstanding and disconnection if unrecognized.

The language used to communicate neuroscientific models may additionally contribute to stigmatization through the recruitment of unexamined associated biases, with clinical ethnographic and qualitative evidence identifying the complex manner in which neurobiological models are experienced and employed by different groups and in different settings to both positive and negative effects (58, 59). For some patients, conceptualizing the mind as a personal computer may elicit feelings of alienation and dehumanization. For others, neuroscientific explanations of psychiatric illness may suggest inalterable biological determinism or “neuroessentialism,” contributing to greater prognostic pessimism (30, 60). In one study of mood disorders, neurobiological explanations through neuroimaging offered several patients a sense of validation and hope for earlier or better treatment, with some perceiving these explanations as reducing blame and others’ fears, and yet this framing of their disorders as more authoritatively “objective” also risked making culturally associated moral judgments about mental illness even more difficult to challenge (58). Therefore, objective, neuroscientific language, even when accepted by the patient, may limit how the patient discloses their personal experience. More speculatively, it could work to undermine psychological change.

Moreover, brain-circuit descriptions leverage cultural trends that emphasize the power and sophistication of computer engineering, “hacking,” and artificial intelligence. Such language may reinforce the power differential between the psychiatrist (the powerful technician) and the patient. Scientific language suggesting explanatory certainty further emphasizes the authority of the provider and the promise of their treatment. The physician’s provision of this technical language has a powerful and propagatory effect: the physician provides their precise *expert system* to the patient, who then incorporates a subset of the ideas provided into their personal *explanatory system*, and as resultant explanatory systems become more widely known they are adopted by the lay population as *common sense* (61). In this manner, technical medical language can reinforce stereotypes and power dynamics during clinical visits (62) and has been used historically as a cover to advance racist, sexist, and homophobic ideologies (63).

It is apparent that provider-patient communication through neurobiological models and language is counterintuitively often associated with heightened stigma and a reduction in provider empathy. We have outlined several possible reasons for these findings, including a perception of neuro-determinism, use of language that accentuates the power differential between provider and patient, and the use of impoverished, but objective, biological metaphors. To place this hypothesis in more severe terms: neurobiological metaphors evoke a model of mental illness and mental life that risks foreclosing the relevance of individual subjectivity and creative change, to the potential detriment of clinical recovery. To conceptually analyze this hypothesis, we must examine how metaphors are created in the domains of clinical science and clinical practice. In particular, we will examine two contrasting (although not mutually exclusive) intentions or goals of metaphor creation and communication: (1) a clarifying, “objective” goal, and (2) an evocative, “subjective” goal. To examine the process of metaphor creation we utilize the framework of Peircean semiotics, which is particularly well-suited to examining the unique, subjective dimension to metaphor creation. By identifying the process of metaphor creation (and interpretation), clinicians stand a better chance of aligning their communication with the relevant audience.

## Metaphor and evocative intent

In communicating metaphors of mental illness, we share our models of the mind. When a patient names depression as “my demons,” this metaphor for their illness reflects a system of understanding, in other words, a model of illness. Moreover, we consider that the metaphors used by patients and those used by clinicians may arise in the context of differing goals or intentions. By exploring the semiotic process whereby metaphors are created and used, we can come to a better understanding of our clinical and neurobiological models of mental illness. We argue that metaphor creation can mirror scientific reasoning and/or poetic logic, as well as clinical reasoning insofar as it draws from both. By poetic logic, we mean the creative, literary, or rhetorical approach, dating back to at least Aristotle (64), which is distinct from scientific reasoning (as we will discuss below). Metaphors, while often novel and creative, also reflect associative learning, contextual significance, and hypothesis generation, all of which are core aspects of clinical practice. Rather than finding a single “right” metaphor, be it neurobiological or otherwise, we will consider how the ongoing process of metaphor creation and co-elaboration may be of novel value to clinicians.

Non-literal, metaphorical language is frequently used to describe mental illness by patients, psychiatrists, and even the Diagnostic and Statistical Manual of Mental Disorders (65). Patients frequently invoke metaphors when discussing psychiatric symptoms, particularly in conversations outside the clinician’s office such as in casual and online communication. These metaphorical expressions often reflect patients’ cultures, and though some common metaphors cross many distinct cultures, others may be highly specific or subtle (66, 67). Coll-Florit and associates reviewed blogs about mental illness and used qualitative analysis to categorize the posted metaphors (68). Broadly, they found that metaphors represent psychiatric disorders as *living*, *dynamic*, or *static*. In terms of frequency of use, “living organism” accounted for 30% of metaphors, which was further divided into three subcategories: a monster/an evil being/a ghost, a person with whom you live, or something to which you attribute characteristic human actions. Beyond “living organism,” the next most common metaphors included “descent” (~17%); “thing” (~11%), “weight” (~10%), and “unbalance” (9%), followed by less frequently used metaphorical domains. The common use of *living*-themed metaphors highlights the potential for incongruence between patients’ lived experiences and the chemical imbalance or circuit-based descriptions, as these latter metaphors largely draw upon reductionist and inorganic associations. Interestingly, in contrast to the patients’ frequent use of *living*-themed metaphors, and despite the fact that biology is a “life” science, many biologists tend to employ non-living and reductionist explanations for phenomena. This is, in some sense, an unfair contrast; it is likely that the intention of metaphor use by patients and clinician-scientists is fundamentally different.

Within the realm of science, metaphor and its cousin, analogy, are often employed not to amplify literary themes but rather to achieve greater clarity. There is a long history of scientists making explicit use of analogical reasoning to improve clarity of thought and to build new hypotheses (69). Analogical reasoning can be contrasted with older approaches within science, epitomized by alchemy, where evocative amplification was the goal. As described by Gentner and Jeziorski, alchemical use of “unruly metaphors” from “surface similarity” and “richly interconnected but unclarified forms of similarity” are quite at home in the domain of art and creative literature (69). They suggest that the alchemists had different goals from today’s “logical” scientists, that is, concerned with personal and spiritual matters that are typically separate from the scientific enterprise. This example provides an illustrative framework for the ways that metaphors are created, intended and interpreted. When Rosenman states that with respect to psychiatry “metaphor may not only amplify, it may also constrict the ways that we frame a particular concept” (56), we suspect he is pointing toward these two poles of how metaphors (language, and signs more broadly) come to be understood through semiosis. Constricted framings might in fact be quite helpful if one is trying to come to a communal understanding, satisfying a goal of more “objective” clarity at the potential expense of “subjective” creativity.

The landmark work *Metaphors We Live By* (70) and subsequent elaborations into Conceptual Metaphor Theory (CMT) firmly established a cognitivist framework describing conceptual metaphor as “a systematic set of correspondences between two domains of experience” (16). Further speaking to this ability of metaphors to draw from varied domains, it has been argued that there is no single “module” for metaphor or abstract thought within the brain, and that metaphors instead recruit and link diverse areas of the brain in complex “cascades” (71). To the extent that human experience can be understood through shared embodiment, CMT helps identify primary and universal metaphors. In this way, CMT supports the thesis that in some sense, all cognition is metaphorical reasoning. Further, it illustrates how the deployment of metaphorical language in the scientific process benefits from an underlying conceptual architecture. Nonetheless, the broad use of the term metaphor also points toward the domain of the creative arts, which for some is not well captured by CMT (72, 73) nor necessarily dependent on conceptual relationships, more broadly understood. Although creativity is not incompatible with CMT, metaphorical creativity was not necessarily the focus of the earlier forms of the theory (16).

When the field of psychiatry is criticized for using “chronically anemic” language and clinicians are suggested to explicitly offer their patients “a dose of metaphor” (65), we suspect that these “creative’’ or poetic domains are being evoked. Peircean semiotics offers a useful framework for capturing the distinction between personal/subjective and communal/objective poles (74). For Peirce, the process of semiosis was interpretive and subjective. Peirce posited that sign relations reflected the triadic dynamics of an object, its sign, *and the interpretant* (the sense produced in the mind of a subject). Sign relations are further categorized as iconic, indexical or symbolic. Briefly, icons are based on similarity and resemblance, indices are based on correlation or connection to something else, and symbols are based on social convention. In some sense, anything can be an icon or an index depending on the intention, but symbolic function and language is dependent upon human brains and sociality (75, 76).

Relevant to our discussion is the semiotic basis for metaphor, discussed at length by many semioticians (72, 77, 78). Metaphor depends on varied and rich subjective interpretations and iconicity in particular (72, 79). This fosters “unlimited semiosis” (80), a “creative process without restriction” (72), based on underlying similarity that can be novel or idiosyncratic to the individual. Metaphors emergent from this process feel “fresh” and “alive,” mirroring the unbounded creativity and iterative dynamics inherent in biology and the evolutionary process (81, 82). A process of semiotic evolution (79) proposes how metaphors become overused and, in effect, are no longer metaphors; that is, they become conventional symbols. In this way, brain circuits and chemical imbalances have lost their original metaphoric novelty and now serve as symbols for mental processes. Such repeated and highly-specified metaphors have been referred to as “dead metaphors” (57, 83, 84) or “historical metaphors” (85) that no longer elicit evocative creativity.

Moreover, metaphoric logic, that is *iconic logic*, is not limited to language proper. Susanne Langer described how art and music are conveyed iconically, using the term “presentational” to relate the image and its iconic essence (86). As she further explained, “a metaphor is not language, it is an idea expressed by language, an idea that in its turn functions as a symbol to express something. It is not discursive and therefore does not really make a statement of the idea it conveys; but it formulates a new conception for our direct imaginative grasp” (87).^2^ Thus, metaphoric processes can be independent of language and might be pre-conceptual, reflecting dynamical iconic relations that are particularly consistent with both musical elaboration and the modular organization of the cerebral cortex (88). The key is what the metaphor *expresses*, which according to Langer and for the arts, is emotive forms. In this sense, iconic reasoning is a form of emotive reasoning in the broadest sense of felt reasoning (well-exemplified by the colloquial phrase “I feel you” to express shared understanding). Thus, metaphors based on this iconic-emotive reasoning are personal, but can be inter-subjective. Longstanding therapeutic relationships begin to develop their own language, as in language style matching (89) that could be based on fundamental languaging dynamics (90). Personal metaphors “cohere” to experiences in life in a way that facilitates experiential learning and self-understanding. Through psychotherapy, the construction of new coherences in life gives new meaning to old experiences.^3^ The therapist and the patient co-author the formation of personalized metaphors of the patient’s life to make sense of the unknown or intolerable. This shared generative process exemplifies the value of novel co-elaborated, personalized, *living* metaphors.

## Personalized metaphor and clinical reasoning

Given these varied meanings, uses, and understandings of metaphor, it is no surprise that patients, caregivers, and medical professionals use a wide range of metaphors (91). For example, the metaphors of “journey” and “war” are frequently employed in the context of oncologic illness but vary in their significance between patients. For instance, one patient may feel comforted by a metaphor of being on a “cancer journey” with a sense of hope and progress, though another may find the idea of a journey as anxiety-provoking and demoralizing. Similarly, violence metaphors can be seen as assigning blame and failure for some patients, while others may find that they provide a sense of purpose and self-determination. This variety of metaphors is also found in other conditions, with such themes as “journey,” “transmittable,” being an “object,” or being “person-like” used for hypertension and diabetes (92). Others have suggested potential pitfalls when using value-laden metaphors, such as “broken heart,” for stress cardiomyopathy, where the etiologic foundation and treatment approach remain in flux (93). Accordingly, not all studies demonstrate a preference for metaphoric language (94), again highlighting that the ways in which metaphors and language are used clinically may have very different implications for different patients and must be personalized.^^4^^

This importance of personalized metaphors has led some clinicians to encourage the use of adopting a “menu” of metaphors that can be readily accessed and offered to patients (96). Intentionally finding language to co-author an experience in this way can therefore help develop personalized meaning, rather than relying on the clinician’s application of the “right” metaphor or narrative. Furthermore, deficits in *non-literal* language comprehension are prominent features of many psychiatric disorders, including schizophrenia and autism spectrum disorder (97, 98). Treatments to improve metaphorical discourse, where the clinician actively works with the patient to develop the language used to describe their experience, may be a particularly valuable therapeutic target in such cases. There is considerable research on different methods of effectively using metaphors in clinical practice, including its applications to numerous psychotherapeutic modalities and medication management settings (99–101), as well as randomized controlled trials across different populations and a variety of medical conditions (102–104). More broadly, possible existing clinical approaches have included working from the patient’s own metaphors (such as changing a patient’s own “metaphorical kernel statement”) or introducing novel clinician-generated metaphors which are then co-elaborated (105, 106).

Separate from establishing a new set or “menu” of specific metaphors, a potential clinical advantage of the evocative process of novel metaphor generation is its invocation of *metaphorical thinking* by promoting *abductive reasoning* as a means of understanding mental illness. Abduction is a term from Peircean semiotics, outlining the process of associative reasoning whereby a hypothesis is formed from existing facts. As distinct from deductive or inductive logic, abduction need not rely on conceptual mechanisms and can be established through iconicity (107). That is, abduction has been used to explain both logical forms of hypothesis testing and creative or imaginative reasoning. Metaphor and abductive reasoning facilitate the learning of unfamiliar meaning from contextual association (81, 108), and a common cognitive mechanism has been theorized to underlie both abductive logic and metaphors (109). Clinically, the role of metaphor generation and abductive reasoning may allow clinicians and patients to see the diagnosis from a new “way of seeing” (110). Therefore, metaphoric thinking may be of benefit to patient and clinician in an effort to more fully understand mental illness, and the unique experience of mental illness in an individual. While there have been efforts to apply Peirce’s approach more specifically to psychiatric practice (111), its use remains limited within medicine more broadly (112–115). Indeed, clinical research has not often emphasized the creative aspect of abduction (116), even though this creative aspect may reflect intuitive and imaginative reasoning which are of clear importance to science and medicine (117, 118).

Of note, the creative and poetic intent of metaphor in conveying the experience of mental life both predates and continues alongside modern science in literary descriptions. Congruent with abductive logic, living metaphors often depict hypotheses, using prior experience to understand, anticipate, and evoke a novel context or understanding. When Emily Dickinson describes *despair* as being “…Without a Chance, or spar –/Or even a Report of Land –,” she invites, as a possibility (“even”), the image of being lost at sea without any ship or coast that might provide a means to survive (119). Through this broader contextual association, the reader can begin to explore the implications of this despair: a profound hopelessness, an endless solitude, with no sign of relief or salvation. Classic, literary descriptions such as this evoke the meaning of despair in a manner that invites active interpretation (hypothesizing) by the reader grounded in their own individual associations. In a more modern example, philosopher David Abram exemplifies one approach of embracing living metaphors when describing the mind, likening thoughts to various animals within a complex ecosystem:

*[C]ertain ideas were like deer … graceful, shy, lingering at the edge of our awareness, yet slipping back into the forest if too willfully focused upon … Some of them slither mostly unseen through the grass … others bask on warm rocks in the midafternoon, only to skitter away at our incautious approach. Certain lightweight thoughts flutter in the air around us, so small and erratic we easily neglect to notice them, while other more muscled notions lope unexpected across our roads, marking their passage with scent or scat* (120).

The mind as an ecosystem is just one example of living metaphors, evoking concepts of *dynamis*, diversity, and interconnectedness, without imposing a specific interpretation or valence. Such metaphorical language is *living* in two ways: both in its evocation of a living ecosystem, and its impressionistic quality that is not overly-specified (in contrast to a “dead metaphor”) and therefore shapeable to its *interpretant*. An emotional landscape likened to a forest may represent a fearful loneliness for one and a vibrant solace for another.^5^ In this way, metaphorical language and abductive reasoning afford a powerful approach toward capturing a concept’s uniqueness and contextual dependence. Living metaphors are inherently creative and collaborative: in being *non-literal*, they demand that listeners recruit their individual associations to interpret the concept being communicated, thereby enabling the co-authoring of shared meaning. As such, poetic metaphors may offer a valuable mechanism where providers’ *expert systems* and patients’ *explanatory systems* meet to creatively conceptualize personalized models not directly derived from existing frameworks.

Caution is required in this undertaking: as valuable as metaphor may be for communication and generative conceptualization, there are times when even the best-intentioned language inevitably falls short. A new metaphor may not always be better than the last at cultivating flexibility, abductive reasoning, tolerance, clarity, and positive change. Unexamined, a poorly chosen novel metaphor may counterproductively convey misleading certainty or coherence, or recruit harmful biases. Ultimately, the use of *any* language to describe aspects of mental illness might be off-putting for some (56). In fact, the shift to verbal reasoning may undermine insight (124). This idea relates to the importance of *non-verbal* languaging (125) and staying silent when there are no words to capture the experience of illness (126). Hence the application of metaphor and narrative is not about finding the “right words,” since metaphor is, by definition, not literal. Rather, the abductive process of searching for new words – and, as needed, silences – may be part of the therapeutic process. In this manner, the application of metaphor begins to align more closely with Langer’s feeling and iconicity, that may be pre-conceptual, related to the cadence of speech as much as the content. It also acknowledges the inherent ambiguity and uncertainty in metaphor. This uncertainty was present in Peirce’s related doctrine of fallibilism in the semiotic process, the willingness to be mistaken and subject the process to ongoing inquiry and evolution (127). Acknowledging the role of this uncertainty in practice, it may be valuable for clinicians to purposefully cultivate metaphors that preserve elements of ambiguity (fitted to patient and context). Similar to open hypotheses, such metaphors are malleable, open to correction and re-imagination, and can thereby mature into personalized metaphors that embody the same potential for change and growth we wish to encourage in our patients.

## Conclusions: neurobiology, metaphor, and uncertainty

Mental health clinicians are challenged to explain the etiology of illnesses that lack consensus scientific understanding. In the above analysis, we have reviewed evidence that the dominant explanatory model of mental illness, the neurobiological model, can have adverse effects when communicated to patients and to the public at large. These effects include inadvertently worsening stigma, reducing provider empathy, and undermining public trust. In part, these effects may be due to the metaphorical implications of commonly-employed neurobiological language. Subsequently, we reviewed the role of metaphor in scientific and clinical practice. Our analysis has outlined how metaphors play multiple simultaneous roles in the conceptualization and communication of psychiatric illness, including the dual goals of (1) achieving clearer, more restrictedly-defined shared understandings, and (2) evoking creative and at times novel meanings. We have also identified how the process of languaging metaphor overlaps with abductive clinical and scientific reasoning, since they rely on prior experience, employ associative logic, and reflect an estimation of reality. In this concluding section, we extend our position to highlight the importance of *uncertainty* as a key feature of metaphors that are flexible, evocative, and personalizable, and how the process of metaphor creation is a powerful tool for both engendering and communicating the broader etiologic uncertainties within psychiatry.

Our analysis has attempted to draw attention to both the process of metaphor construction and the evocative content that a metaphor generates. Models such as chemical or circuit imbalance might be used when an appearance of greater explanatory certainty is desired; these descriptions evoke a broad model of brain function that is precise like a computer, or suggest chemical reactions that fit “lock and key.” Thus, they seem to evoke the rigor of science. However, in addition to the shortcomings of computational descriptors that we discussed above, these descriptors emphasize the material parts and their static nature. Newer emerging models of brain function emphasize dynamic systems that suggest more resonance with other living systems, or even ecosystems. Hence the brain has been likened to a complex orchestra (128), one that balances a well-worn score with improvisation, instilling novelty within a developmental and evolutionary trajectory. Moreover, that the brain itself may be operating semiotically, that is via interpretive dynamics within networks, illustrates a potentially foundational connection between metaphor, neurobiology and the creative arts (88). In other words, current theories of Peircean semiotics that highlight the subjective-interpretive, the poetic-iconic, and the doctrine of fallibilism are not merely frameworks for the humanities, but inform neurobiological processes as well (129, 130). Given this, clinicians can play an active role in languaging new metaphors based on their and their patients’ experiences to assist translational cognitive neuroscience in formulating new models of brain function and illness. More work is needed to bridge neurobiological mechanisms and biosemiotic theories, but the clinical sciences could be fertile ground for this dialogue. A twofold metaphorical perspective may help relieve neurobiological models of the pressure of having to provide a single, and final, “ground truth” and, in so doing, support greater uncertainty tolerance.

Clinically, uncertainty and ambiguity might be considered central tenets of psychiatry (131). Metaphors that maintain elements of ambiguity (which are not overly-restricted or calcified in their meaning) are not bound to the literal, and so can maintain flexibility over time. Building upon the premise of cognitive flexibility as an important component of healing that may be promoted through metaphoric transformations (132), flexible metaphorical language and its recruitment of abductive reasoning poses potential advantages in both communication and complex clinical decision-making. More speculatively, metaphorical language that allows for ambiguity may support the development of greater uncertainty tolerance in clinical encounters. Given increased attention on how to better understand and develop uncertainty tolerance in clinicians (133, 134), metaphor generation and co-elaboration as a metacognitive process may be one promising avenue for uncertainty tolerance development in trainees and practicing clinicians alike.

We have aimed to demonstrate why the active process of metaphor creation and co-elaboration satisfies aspirations for a more effective language in communicating psychiatric pathology. Metaphors carry the two separate goals of establishing shared, more “objective” clarity, and evoking flexible, more “subjective” creativity. However, even initially rich metaphors can, as they are further reinforced and defined, become restricted and stagnant over time. Though these calcified metaphors may meet the first goal of a clearer shared understanding, in their fixedness they lose the potential to meet the second goal of evocative creativity and potential for change. Metaphor generation is distinctly important because, in the abductive, generative process, the seeking of novel metaphors (hypotheses) preserves the second goal. They maintain an element of the undefined, the uncertain, and yet the possible. This does not relieve us of the responsibility to find and use metaphors (or treatments, for that matter) tailored to purpose, and any specific metaphor may be helpful or harmful depending on how it is applied. Sometimes the certainty of a more tightly-defined metaphor may be needed to reassure a patient, and at other times a more ambiguous metaphor requiring uncertainty tolerance may push a different patient to find new, more helpful meanings. But more broadly as a meta-process, as metaphors are allowed to change, so can our personal and societal understanding and beliefs. This ability to continually shift conceptualizations through metaphor generation and metaphorical thinking is important to expand the capacity for patient recovery and reduction of stigma.

This conceptual analysis has several limitations. Peircean semiotic terms are not commonly employed within clinical research, are interpreted inconsistently, and frequently do not have a shared meaning between disciplines. Compounding these challenges is the limited evidence base for what type of language, in practice, appears to be most clinically valuable with patients. To our knowledge, although the feasible implementation of metaphorical language has been explored in some modalities, such as in Acceptance and Commitment Therapy (135), more research is needed into the systematic use of metaphorical language across multiple practice settings, including within psychopharmacology-focused visits. Given the variability in how “metaphors” are defined and their measures operationalized between studies, there is significant opportunity for identifying clearly what is meant by “metaphor” within a given study to enable more appropriate comparison and synthesis between studies. With regard to clinical decision-making, future studies might examine whether metaphorical speech correlates with the use of abductive thought and related cognitive processes, or is associated with differences in uncertainty tolerance. It may also be fruitful to investigate the feasibility and outcomes pertaining to the directed use of metaphorical language in clinical practice and in medical education. Though it is outside the scope of this analysis, further conceptual work might consider a synthesis of these topics in the context of work in narrative medicine, given the centrality of illness models (and the specific language recruited to describe them) in patient and provider narratives.

As a result of their training and clinical practice, many psychiatrists are often highly skilled in metaphorical communication and reasoning (136). Psychiatrists are also already particularly attuned to the importance of holding uncertainty, engendering not only tolerance but curiosity for their patients through this holding. Therefore, the co-creation of dynamic metaphors with patients in a more intentional, self-reflective, focused, and creative manner is a feasible approach to further study. The formal practice of tracking what metaphors are being used by patients, and consideration of which are of greatest therapeutic value, may be of high potential impact even in time-limited medication management settings. By remaining ever-present to the subjective experiences of their patients, their narratives, and metaphors, mental health providers will always be challenged to balance this perspective with mechanistic models. As the psychiatrist, philosopher, and author Iain McGilchrist warns:

*You can so alienate yourself from a poem that you stop seeing the poem at all, and instead come to see in its place just theories, messages and formal tropes; stop hearing the music and hear only tonalities and harmonic shifts; stop seeing the person and see only mechanisms* (118).

Meaningful explanation is found by holding multiple truths in a manner that includes both clarifying and poetic aspects of language use.^6^ We have argued that mechanistic terminology recruited by contemporary advances in neurobiology cannot be simply transposed as convenient descriptors of complex mental processes or individual subjective experience. As so eloquently suggested by philosopher and poet Jan Zwicky in the opening epigraph, our non-metaphorical language – that is, the language of neurobiology – is arguably the necessary foil to facilitate metaphorical reasoning that embodies the human *art* of medicine. Perhaps this approach can influence how we speak to our patients and relieve psychiatry of the need to explain both the mechanism and experience of illness with absolute certainty. As such, by languaging metaphor with intentionality, we can relax our gaze from a static terminology of psychopathology and, instead, evoke workable depictions of mental illness that encourage transformation and recovery.

## Author contributions

AS: Writing – original draft, Writing – review & editing. MJ: Writing – original draft, Writing – review & editing.

## Footnotes

1 We use “languaging” (19) here in the broadest sense possible: as “co-created meanings (that) arise out of linguistic relationships” and define “the ways in which we see the world and how we communicate, but it also limits and confines us” (20).

2 Of note, and perhaps confusingly, Langer uses the term “symbol” in the same manner as semioticians use “sign.”

3 We acknowledge our colleague Fredrick Steele for this particular language.

4 Highlighting the centrality of personalized language, it has been argued that the struggle to make sense of and articulate inner emotions plays a central role in the experience of suffering (95). More speculatively, it has been suggested that suffering itself may arise because of our capacity to reflect or represent our experience through language (53).

5 Regarding the content of this extended metaphor, there is increasing evidence for the therapeutic effect of exposure to nature, whether real or virtual (121–123), although additional research is needed into whether the use of language evocative of natural imagery itself may invite similar benefits.

6 Or as McGilchrist (2021) describes, there is a subtle but relevant etymological distinction between being precise (cut-short, before its time) and accurate (done with care). The objective, clarifying truth typically relies on being precise, whereas the poetic aspects emphasize accuracy to experience.
